# A Retrospective Cohort Study Comparing the Clinical Outcomes of the Hydrosurgery System and Traditional Single‐Incision Surgery for Axillary Osmidrosis

**DOI:** 10.1111/jocd.16755

**Published:** 2025-01-13

**Authors:** Lufan Xia, Mu He, Xiaoqiang Liu, Jinghong Zhang, Ying Chen, Jiaping Zhang

**Affiliations:** ^1^ Department of Plastic Surgery, Southwest Hospital Third Military Medical University (Army Medical University) Chongqing China; ^2^ Department of Plastic Surgery The Second Hospital & Clinical Medical School, Lanzhou University Lanzhou Gansu China

**Keywords:** axillary osmidrosis, axillary osmidrosis surgery, Hydrosurgery, hydrosurgery system

## Abstract

**Purpose:**

To compare the postoperative scarring, complication rates, and efficacy between the hydrosurgery system and traditional single‐incision surgical techniques for treating axillary osmidrosis.

**Methods:**

A retrospective collection was conducted of all patients who underwent radical surgery for axillary osmidrosis at the Day Surgery Unit of the Department of Plastic Surgery at the First Affiliated Hospital of the Army Medical University from January 2023 to January 2024. Patients were screened based on inclusion and exclusion criteria and divided into the hydrosurgery group and the traditional surgery group. The evaluation was done through medical records, follow‐up records, electronic questionnaires, and photographs. Assessments included 6‐month postoperative scarring conditions, comprehensive effectiveness scores, postoperative odor/hair/sweating scores, postoperative dermatological life quality scores, and complication rates.

**Results:**

A total of 73 patients completed this study: 34 in the hydrosurgery group and 39 in the traditional surgery group. 6 months postoperatively, the hydrosurgery group had significantly lower scores of the Vancouver Scar Scale (VSS), 0.5 (range 0.0–2.0) compared with 1.5 (range 0.5–3.0) in the traditional surgery group (*p =* 0.018). The incidence of complications such as subcutaneous hematoma, epidermal necrosis, and wound dissidence was also lower (26.5% vs. 51.3%, *p =* 0.031). Additionally, the surgical incision length in the hydrosurgery group was significantly smaller than in the traditional surgery group (1.200 cm (1.000, 1.275) vs. 2.500 cm (2.500, 3.000), *p* < 0.001). There were no significant differences between the two groups regarding comprehensive effectiveness scores, postoperative odor/hair/sweating scores, postoperative dermatological life quality index, and surgical duration.

**Conclusion:**

Compared with traditional single‐incision surgical techniques, patients in the hydrosurgery group exhibited lower scar scores 6 months postsurgery, required smaller surgical incisions, and had lower rates of surgical complications. Thus, the hydrosurgery is suitable for the minimally invasive surgical treatment of axillary osmidrosis, offering a safe, effective, and aesthetically superior treatment method.

## 1 Introduction

The interaction between apocrine gland secretions and bacteria in the axillary region causes axillary osmidrosis, resulting in an unpleasant odor [1]. It often appears after puberty and has a clear genetic predisposition. Moreover, it significantly impacts patients' social interactions and quality of life. Current treatments for axillary osmidrosis include surgical and nonsurgical methods. Traditional surgery, involving subcutaneous trimming to remove apocrine glands, is the mainstream choice due to its reliability and low recurrence rate. Still, it usually chooses 1–2 incisions about 3–4 cm long, thus resulting in significant scarring and requiring high surgical skill and experience [2, 3, 4]. Nonsurgical treatments include lasers, radiofrequency [5], microwaves [6], absolute ethanol injections [7], botulinum toxin type A injection [8] and topical medications [9, 10]. While these methods are less invasive and easier to perform, they do not completely remove apocrine glands, making their effects temporary, and necessitating repeated treatments. The emergence of minimally invasive techniques such as suction‐assisted lipectomy, mechanical liposuction, and ultrasonic liposuction has resulted in lower complication rates than traditional surgery; however, they do not effectively remove centrally located apocrine glands in the axilla, potentially leading to higher recurrence rates [11, 12, 13]. Thus, achieving complete odor removal while minimizing surgical trauma and scar formation remains a significant challenge in the surgical treatment of axillary osmidrosis.

The hydrosurgery system, a new surgical tool, produces micrometer‐sized supersonic water jets, utilizing the Venturi effect and the stress–strain differences in human tissues to perform selective tissue cutting. It has been widely used in soft tissue debridement procedures such as burn wounds and chronic ulcers [14, 15, 16, 17, 18, 19]. In 2013, Korean researchers first applied the hydrosurgery system to treat axillary osmidrosis, with clinical studies indicating ease of operation and high patient satisfaction [20]. However, studies comparing the effects on postoperative scarring and complication rates between the hydrosurgery system and traditional surgery show mixed results. Furthermore, research suggests that using a single‐incision technique with the hydrosurgery system is safer than traditional double‐incision methods, but comparative studies are scarce [21]. Therefore, this retrospective cohort study was designed to evaluate the differences in postoperative scarring, complication rates, surgical efficacy, and patient satisfaction between the hydrosurgery system and traditional single‐incision surgery, provide a reference for clinicians at the same time.

## 2 Patients and Methods

### 2.1 Study Design

This study is a retrospective cohort study including all adult patients who underwent radical surgery for axillary osmidrosis at the Day Surgery Unit of the Department of Plastic Surgery at the First Affiliated Hospital of the Army Medical University from January 2023 to January 2024. Patients were assigned to the hydrosurgery group (HS group) or the traditional surgery group (TS group) based on the surgical technique. Medical records, clinical visit notes, and routine follow‐up records were reviewed to collect demographic and clinical data. Patients were excluded if they had undergone previous axillary odor surgery or received botulinum toxin injections, laser, or radiofrequency treatments for axillary osmidrosis up to 6 months before admission; were pregnant or breastfeeding women; were undergoing or had undergone systemic immunosuppressive therapy (including corticosteroids), cytotoxic drugs, or anticoagulant therapy; had diabetes, hypertension, coronary heart disease, vascular diseases (including un‐reconstructed peripheral vascular disease, and vasculitis), pyoderma, or lymphedema; had acute or chronic bacterial, viral, or fungal skin infections potentially interfering with wound healing; had a body mass index (BMI) ≤ 18.5 kg/m^2 or > 30 kg/m^2; were under 18 or over 65 years of age; or had incomplete follow‐up data.

### 2.2 Measures

The primary outcome measure was the condition of the surgical scar at 6 months postoperatively, independently assessed by two full‐time plastic surgeons using the Vancouver Scar Scale (VSS, Table S1), with the average of their scores taken as the final score.

Secondary outcome measures included:

- Complication rate: Defined as the number of patients experiencing complications divided by the total number of patients, expressed as a percentage. Known complications included epidermal necrosis, subcutaneous hematoma, wound dehiscence, recrudescence, and surgical site infection.
- Surgical effect: Evaluated through the Dermatology Life Quality Index (DLQI, Table S2), reduction in axillary odor, hair, and sweating, and overall patient satisfaction. The DLQI score is composed of 10 items (each scoring 0–3), with a total score ranging from 0–30 where a higher score indicates greater impact on quality of life. Odor/hair/sweating reduction was assessed on a scale from 0 (no reduction) to 10 (equal to or worse than preoperative levels). Overall satisfaction was assessed using a comprehensive effectiveness score (Table S3), based on surgical tolerance, complete healing time of the surgical area, improvement in axillary odor, subjective evaluation of scarring, and shoulder joint mobility, each rated on a scale of 1–3 points.

### 2.3 Procedures

**FIGURE 1:**
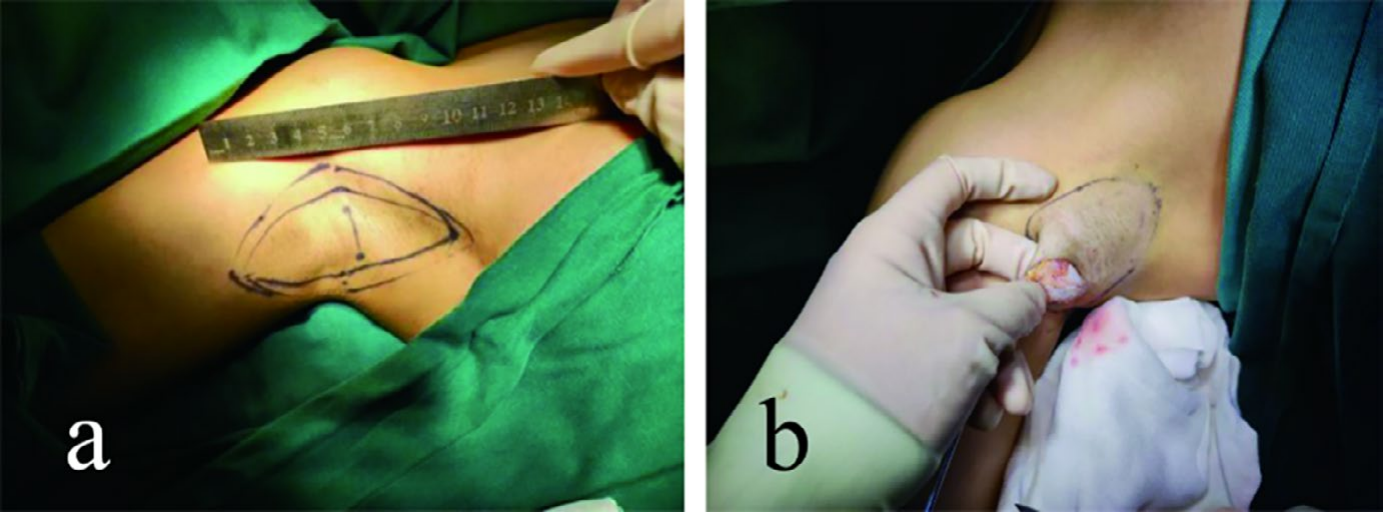
Mark the surgical area (a) and flip the skin to check (b).

All surgeries were performed under local anesthesia, adhering to standard clinical practices. Except for the specific surgical technique, all other surgical‐related elements were identical between the groups (Figure 1a,b). To be specific:

#### 2.3.1 HS Group

- The patient was positioned supine with both upper limbs abducted at 90°. Bilateral upper arms, axillae, and chest were disinfected using 0.5% povidone‐iodine, and sterile drapes were applied to expose both axillae.
- The range of axillary hair was measured and documented. A subcutaneous dissection area was marked 1 cm beyond the edge of the axillary hair. An incision was designed along the skin crease, and photographs were taken for documentation.
- Local tumescent anesthesia was administered to the marked area using 0.25% lidocaine with 1:1000000 epinephrine. The anesthetic solution was allowed to infiltrate for 10 min.
- A No. 15 scalpel was used to incise the skin, and surgical scissors were employed to separate the subcutaneous space within the superficial fascia layer, creating a flap approximately 1 cm thick.
- The hydrosurgery system (*Hydro CareSYS I, HYDROCARESYS Medical Co., China*) was connected. The operating level was adjusted to settings 3–5 based on intraoperative conditions. The treatment window of the system was directed toward the dermal surface. Repeated fan‐shaped movements were performed to remove fat, apocrine glands, and eccrine glands. Clearance was assessed by palpating the skin and visual inspection through the incision, ensuring a smooth dermal surface.
- The surgical cavity was irrigated with normal saline, and hemostasis was achieved using bipolar electrocoagulation. Small perforations were made in the flap for drainage, and the incision was closed with 5–0 nylon sutures in a full‐thickness closure.
- Postoperative compression of the axillary area was applied using packing and an elastic bandage in a figure‐eight wrap.
- On postoperative Day 7, the dressing was changed. Compression and elastic bandage wrapping were continued.
- Sutures were removed 10–14 days postoperatively. After suture removal, no further compression or immobilization was required.

#### 2.3.2 TS Group

a‐d and f‐i are identical to those in the HS group.

e. The inner side of the flap was everted manually, and scissors were used to remove fat, apocrine glands, and eccrine glands. Clearance was assessed by palpating the skin and direct observation through the incision to ensure a smooth dermal surface.

### 2.4 Statistical Analysis

Normally distributed quantitative data were presented as means ± SD and compared using independent *t*‐tests. Skewed data were presented as medians (p25, p75) and compared using the Mann–Whitney U test. Categorical data were expressed as absolute numbers or percentages and compared using the chi‐squared test or Fisher exact test when appropriate. Rank data were compared using the rank sum test or Mann–Whitney U test. Data analysis was performed using SPSS 27.0 software, and a *p* value < 0.05 was considered statistically significant.

## 3 Results

### 3.1 General Characteristics

A total of 119 patients were treated during the designated period, with 73 meeting the inclusion criteria—34 in the hydrosurgery group and 39 in the traditional surgery group. In the hydrosurgery group, the average age was 24.00 ± 8.72 years, with a female‐to‐male ratio of 7:27. In the traditional surgery group, the average age was 24.62 ± 5.14 years, with a female‐to‐male ratio of 10:29. The groups were well‐balanced in terms of age, gender, BMI, duration of axillary osmidrosis, preoperative grading of axillary osmidrosis, preoperative axillary sweating score, family history of axillary osmidrosis (Table 1).

**TABLE 1:** Baseline characteristics.

Variable	HS group (*n* = 34)	TS group (*n* = 39)	*p*
Age(y)	24.00 ± 8.72	24.62 ± 5.14	0.720
Gender			χ2 = 0.260, *p* = 0.610
Female/male	7/27	10/29	
Height(m)	1.63 ± 0.08	1.64 ± 0.09	0.817
Weight(kg)	59.28 ± 10.52	59.84 ± 12.67	0.837
BMI (kg/m2)	22.03 ± 2.86	22.01 ± 2.88	0.978
Duration(y)	10.41 ± 7.97	9.67 ± 5.19	0.634
Axillary osmidrosis grading (Table S5.)			Z = −0.549, *p* = 0.583
1	2/34 (5.88%)	3/39 (7.69%)	
2	30/34 (88.24%)	31/39 (79.49%)	
3	2/34 (5.88%)	5/39 (12.82%)	
Axillary sweating grade (Table S4.)			Z = −0.692, *p* = 0.489
1	13/34 (38.24%)	17/39 (43.59%)	
2	14/34 (41.18%)	16/39 (41.03%)	
3	3/34 (8.82%)	5/39 (12.82%)	
4	4/34 (11.76%)	1/39 (2.56%)	
Family history	32/34 (94.4%)	35/39 (91.7%)	*p* = 0.679*

^*^Fisher's exact test.

**TABLE 2:** VSS score at 6 months postoperatively.

Scar score	HS group (*n* = 34)	TS group (*n* = 39)	
VSS	0.5 (0.0,2.0)	1.5 (0.5,3.0)	Z = −2.362, *p* = 0.018

### 3.2 Scar Evaluation

**FIGURE 2:**
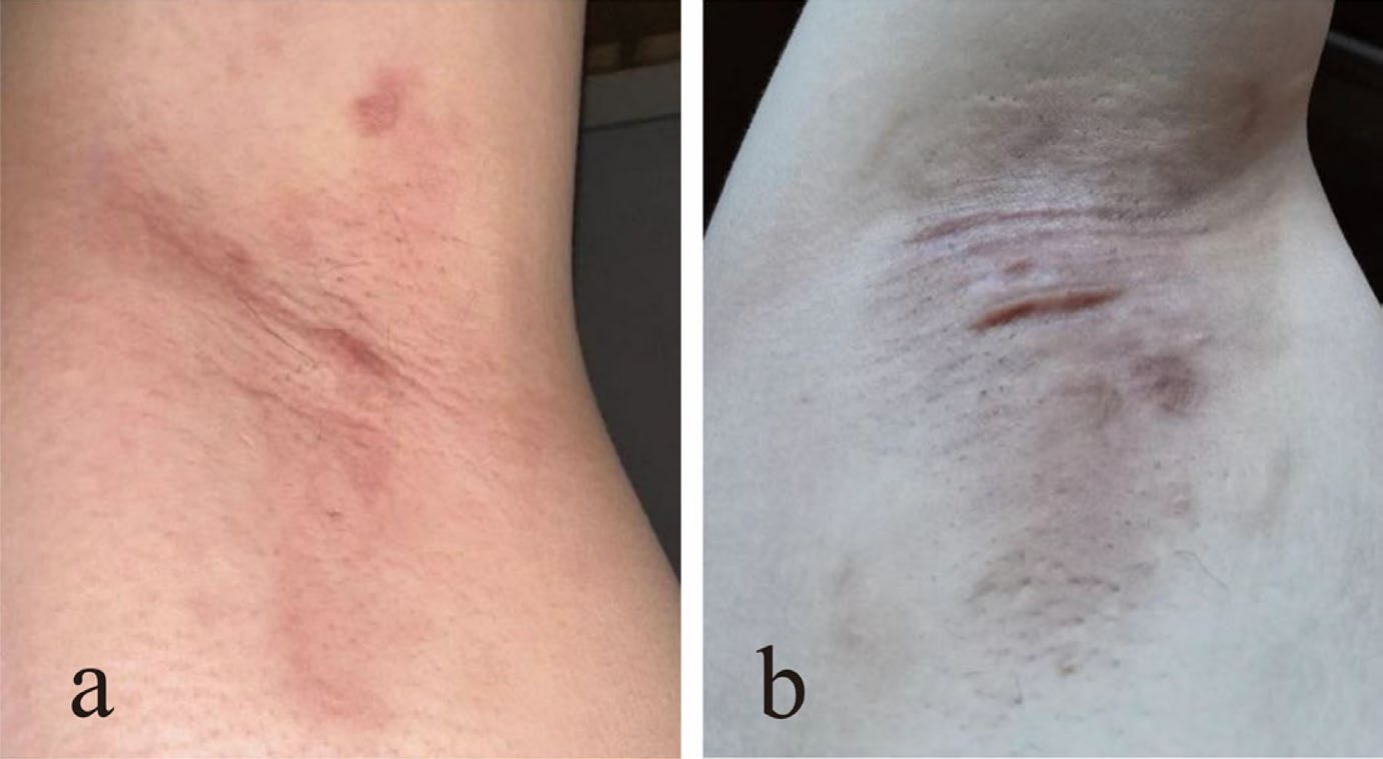
Scar status of the HS group (a) and the TS group (b) at 6 months postoperatively.

Scarring was assessed independently by two professional plastic surgeons outside the surgical team using the VSS scale (Figure 2). The median score in the hydrosurgery group was 0.5 (0.0, 2.0), significantly lower than the traditional surgery group's median score of 1.5 (0.5, 3.0), indicating a statistically significant difference (*p =* 0.018) (Table 2).

### 3.3 Complication Rates

**FIGURE 3:**
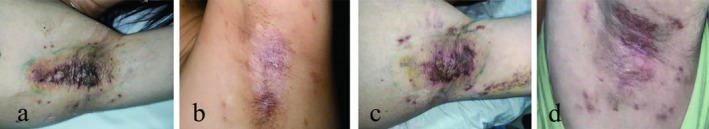
Partial complications and outcomes. Epidermal necrosis in the surgical area 14d (a) and 30d (b) postoperatively; partial incision dehiscence with epidermal necrosis after suture removal 12d (c) and 30d (d) postoperatively.

Differed significantly between the groups, with 9/34 cases in the hydrosurgery group and 20/39 in the traditional surgery group experiencing various postoperative complications including epidermal necrosis, subcutaneous hematoma, surgical site infection, wound dehiscence, and odor recurrence (Table 3). All cases were managed by dressing changes and fully healed on follow‐up, with no cases requiring secondary surgery or grafting (Figure 3). Statistical analysis showed a lower complication rate in the hydrosurgery group (26.5% vs. 51.3%, χ2 = 4.670, *p =* 0.031).

**TABLE 3:** Postoperative complications.

Complication	HS group	TS group	*p*
Hematoma	7/34 (20.59%)	11/39 (28.21%)	0.451
Skin necrosis	6/34 (17.65%)	7/39 (17.95%)	0.973
Infection in the surgical area	0/34 (0%)	1/39 (2.56%)	1.000*
Wound dehiscence	1/34 (2.94%)	2/39 (5.13%)	1.000*
Recrudescence	1/34 (2.94%)	1/39 (2.56%)	1.000*
Total number of people	9/34 (26.47%)	20/39 (51.28%)	χ2 = 4.670, *p = *0.031

^*^Fisher's exact test.

*Note:* Some patients develop multiple complications at the same time.

### 3.4 Curative Effect

Both groups showed reduction in odor, hair, and sweating postoperatively, but no significant differences were observed between them in these reductions. The improvement in life quality postsurgery, as measured by the DLQI score, indicated that both surgical methods effectively mitigated the negative impact of axillary odor on life quality. There was no significant difference between the groups pre‐ and postoperation (Table 4).

**TABLE 4:** Evaluation of surgical efficacy.

Variable	HS group (*n* = 34)	TS group (*n* = 39)	
Axillary sweating score	1.00 (0.00,2.00)	1.00 (0.00,2.00)	Z = −0.139 *p* = 0.889
Axillary hair score	1.00 (0.00,2.00)	1.00 (1.00,3.00)	Z = −0.058 *p* = 0.953
Axillary osmidrosis grading			Z = −1.234 *p* = 0.217
0	12/34 (35.29%)	19/39 (48.72%)	
1	20/34 (58.82%)	19/39 (48.72%)	
2	2/34 (5.88%)	1/39 (2.56%)	
Comprehensive effectiveness score	18/34 (52.94%)	25/39 (64.1%)	χ2 = 0.935 *p* = 0.334
DLQI			
Preoperative DLQI score	4.00 (2.00,8.25)	5.00 (3.00,8.00)	Z = −0.417 *p* = 0.677
Postoperative DLQI score	1.00 (0.00,6.25)	1.00 (0.00,4.00)	Z = −0.120 *p* = 0.904
	Z = −0.2.738 *p* = 0.006*	Z = −0.3.356 *p* < 0.001*	

^*^Wilcoxon signed ranks test.

### 3.5 Satisfaction

The comprehensive effectiveness score assessed 6 months postoperation showed that 18/34 in the hydrosurgery group and 25/39 in the traditional surgery group were rated as having an excellent outcome, with no significant difference in the proportion of patients achieving this rating between the groups (χ2 = 0.935, *p =* 0.334) (Table 4).

### 3.6 Operative Details

**FIGURE 4:**
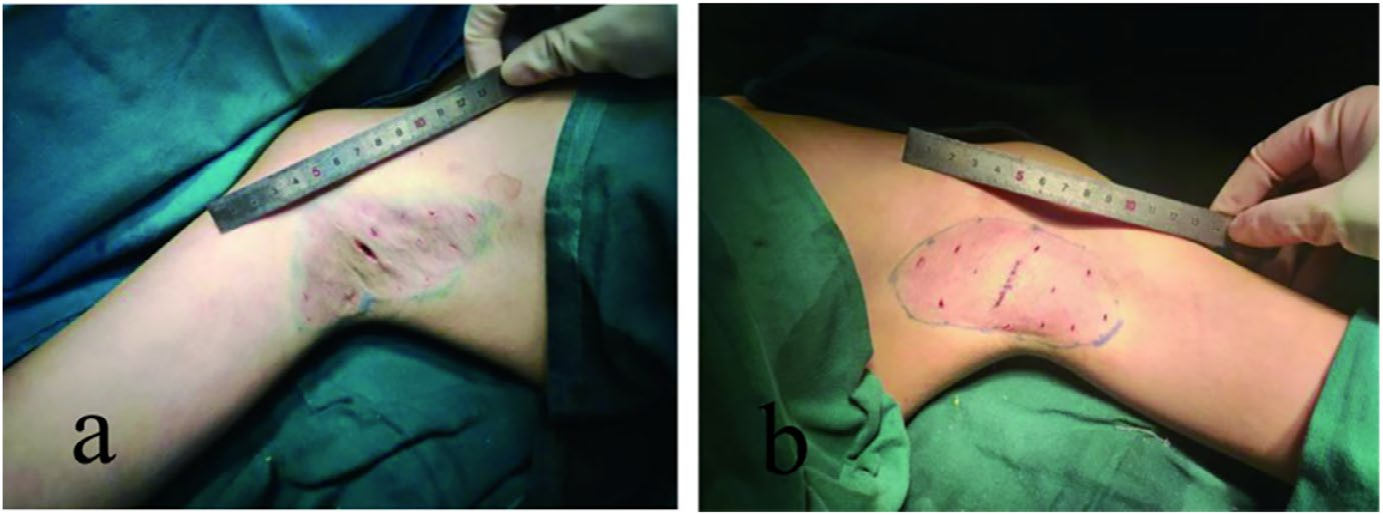
Comparison of the surgical incision length of the HS group (a) and the TS group (b).

The average surgical duration for a single axilla was 114.35 ± 17.764 min in the hydrosurgery group and 112.62 ± 12.030 min in the traditional surgery group, with no significant difference between the groups (Table 5). The median incision length was significantly shorter in the hydrosurgery group (1.2 cm) compared with the traditional surgery group (2.5 cm), and there were no significant differences in estimated blood loss or intraoperative pain between the groups (Figure 4).

**TABLE 5:** Surgery information.

	HS group (*n* = 36)	TS group (*n* = 36)	*p*
Incisive length(cm)	1.200 (1.000, 1.275)	2.500 (2.500, 3.000)	Z = −10.523 *p* < 0.001
Operation time(min)	114.35 ± 17.764	112.62 ± 12.030	0.622
Loss of blood(ml)	10.74 ± 3.008	9.59 ± 2.854	0.100
Intraoperative pain scores (Visual analog scale)	2.71 ± 1.624	2.79 ± 1.625	0.816

## 4 Discussion

This retrospective cohort study evaluates the hydrosurgery system versus traditional single‐incision surgery for treating axillary osmidrosis, focusing on postoperative scarring, safety, and clinical outcomes. Both methods effectively reduced axillary odor, with all patients reporting decreases in hair, sweating, and odor, while maintaining low recurrence rates. Despite the potential of minimally invasive techniques to perform such procedures through smaller incisions, they often fail to completely remove apocrine glands closely attached to the dermis, resulting in higher recurrence rates compared with traditional surgery. The study revealed that both the hydrosurgery and traditional surgery groups significantly improved patients' quality of life, as measured by the DLQI. However, the hydrosurgery group exhibited notably fewer complications and achieved better cosmetic results with lower VSS scores at 6 months postoperatively. The median incision length in the hydrosurgery group was 1.2 cm (range 1.000–1.275 cm), significantly smaller than traditional surgery incisions. These findings underscore the hydrosurgery system's efficacy in achieving comparable therapeutic outcomes to traditional surgery, with the added benefits of fewer complications and improved scar aesthetics.

Six‐month postoperative scar assessments utilized the VSS and were independently evaluated by two plastic surgeons not part of the surgical team, using a blinded assessment approach. Results indicated that the hydrosurgery group had significantly lower VSS scores than the traditional surgery group (*p =* 0.018), suggesting better scar quality in the hydrosurgery group. Consistent with prior studies, the most common postoperative complications in both groups were subcutaneous hematomas or epidermal necrosis, which were fully resolved with conservative dressing changes [20, 22, 23]. Some patients exhibited localized hyperpigmentation, but none experienced significant restrictions in shoulder mobility. Statistical analysis showed that the incidence of postoperative complications was significantly lower in the hydrosurgery group, likely due to the system's tissue selectivity that preserves more subdermal vasculature while removing apocrine glands. This requires further histological confirmation.

Although previous research indicated that the hydrosurgery system could significantly reduce the duration of axillary osmidrosis surgery, this study found no significant difference in operation times between the two groups. Surgeons noted that while gland removal with the hydrosurgery system on one axilla takes only seconds, the small incision limits visibility and makes it challenging and time‐consuming to verify gland removal by turning over the skin. Additionally, the unique design of the hydrosurgical tool may cause unintended skin damage near incision edges, necessitating manual trimming, which extends the surgery duration, likely equalizing the operative times between the groups.

Moreover, this study employed a comprehensive effectiveness score assessing surgical tolerance, complete wound healing time, axillary odor improvement, subjective scarring evaluation, and shoulder mobility (Table S3.). While Xie et al. [22] reported significantly higher efficacy rates in the hydrosurgery group compared to a traditional double‐incision group (83.87% vs. 52.94%, *p =* 0.008), our study demonstrated similar efficacy rates between the hydrosurgery and traditional single‐incision groups (52.94% vs. 63.63%, χ2 = 0.787, *p =* 0.375).

This study has several limitations. It is a single‐center retrospective cohort study with a small sample size, requiring larger, multicenter, randomized controlled trials to validate the results more robustly. Limitations due to follow‐up completeness and medical record availability introduce potential sample biases. Additionally, due to objective constraints, the exact working time of the hydrosurgery system could not be accurately measured, which might affect the comparison of operation durations between the groups. Recommendations have been made to manufacturers to include a timer for the hydrosurgery system's operating time on the control console.

## 5 Conclusion

In conclusion, the hydrosurgery system is as effective as traditional single‐incision surgery in removing axillary odor, reducing sweating and hair growth, and has comparable recurrence rates postoperatively. Additionally, it offers smaller surgical incisions, lower rates of postoperative complications, and better scar healing, proving to be a safe and effective method for treating axillary osmidrosis.

## Author Contributions

Ying Chen and Jiaping Zhang were responsible for the design of the study and the review of the manuscript. Lufan Xia is responsible for data collection, data analysis, and manuscript writing. Mu He is responsible for the coordination of research activities and data collection. Jinghong zhang and Xiaoqiang Liu contributed to the preparation of the article. All authors read and approved the final manuscript.

## Ethics Statement

The study was approved by the Ethics Committee of the First Affiliated Hospital of the Army Medical University. It was conducted by the ethical standards of the institutional and/or national research committee and with the 1964 Helsinki Declaration and its later amendments or comparable ethical standards.

## Conflicts of Interest

The authors declare no conflicts of interest.

## Supporting information


**Data S1**.

## Data Availability

The data that support the findings of this study are available from the corresponding author upon reasonable request.
