# Supplementary material for: A Retrospective Cohort Study Comparing the Clinical Outcomes of the Hydrosurgery System and Traditional Single‐Incision Surgery for Axillary Osmidrosis
Source: J Cosmet Dermatol. 2025 Jan 13;24(1):e16755. doi: 10.1111/jocd.16755 (PMC11726132; doi:10.1111/jocd.16755)
Supplement: Supplementary file 1 — Data S1. [file JOCD-24-e16755-s001.docx]

**Supplementary information.**

**Table S1.The Vancouver Scar Scale（VSS）**

| Project | score | Scoring criteria |
| --- | --- | --- |
| Color |  | 0 points: Scar color is similar to normal compared to other parts of the body |
|  |  | 1 point: Slightly pink |
|  |  | 2 points: Mixed color |
|  |  | 3 points: Dark color |
| Vascularity |  | 0 points: Scar color similar to other parts of the body |
|  |  | 1 point: Pink, slightly higher local blood supply |
|  |  | 2 points: Red, significantly increased local blood supply |
|  |  | 3 points: Purple red or deep red, with abundant blood supply |
| Flexibility |  | 0 points: Normal |
|  |  | 1 point: Soft (able to deform the skin with minimal resistance) |
|  |  | 2 points: Smooth and bendable (able to deform under pressure) |
|  |  | 3 points: Hard (no elasticity when pressed by hand, in block shape) |
|  |  | 4 points: The organization is in a linear pattern (it will retract when stretched) |
|  |  | 5 points: Contracture (permanent shortening of scars leading to functional impairment) |
| Thickness |  | 0 points: Same height as surrounding normal skin |
|  |  | 1 point: ≤ 2mm above normal skin |
|  |  | 2 points: Greater than 2mm and less than 5mm above the surrounding normal skin |
|  |  | 3 points: Greater than 5mm above normal skin in the surrounding area |
| Illustrate | The highest score is 14 points, and the lowest score is 0 points. The higher the score, the more severe the scar condition. Conversely, the lower the score, the less severe the impact. | |

**Table S2. Dermatology quality of life index(DLQI)**

| The purpose of the following questions is to determine to what extent your skin problems are affecting your life. Please only consider the project's situation from last week. | Very serious（3 points） | Serious（2 points） | Lighter（1 points） | None（0 points） | Unrelated |
| --- | --- | --- | --- | --- | --- |
| Are your skin's itching or pain symptoms severe (including soreness, stabbing, etc.)? |  |  |  |  |  |
| How many times have you noticed or felt embarrassed about your skin problems? |  |  |  |  |  |
| To what extent does your skin problem affect shopping, household chores, or gardening activities? |  |  |  |  |  |
| To what extent does your skin condition affect your attire? |  |  |  |  |  |
| To what extent does your skin problem affect your social or leisure activities? |  |  |  |  |  |
| To what extent do your skin problems make sports difficult? |  |  |  |  |  |
| To what extent do your skin problems cause trouble for your peers, close friends, or family? |  |  |  |  |  |
| To what extent do your skin problems cause difficulties in sexual activity? |  |  |  |  |  |
| How big are the problems your skin care has caused you? For example, making the home unclean or wasting time |  |  |  |  |  |
| Does your skin problem affect your work or study? If 'yes', what is the impact? If not, select 'none' |  |  |  |  |  |
| Total score |  | | | | |

**Table S3.Comprehensive effectiveness score**

| Score | 3 | 2 | 1 |
| --- | --- | --- | --- |
| Degree of tolerance during surgery | Light | Tolerable | Untolerable |
| Time to complete wound healing | <10d | 11-20d | >21d |
| Presence of postoperative unpleasant odor | Good | Fair | poor |
| Satisfaction with scars | Great satisfaction | General satisfaction | Dissatisfaction |
| Extent of shoulder movement | Freely | Limited (>270°) | Damaged(<270°) |

**Table S4. Axillary sweating grade**

| Level 1 | I have never noticed sweat under my armpits and it has never interfered with my daily life. |
| --- | --- |
| Level 2 | I can tolerate excessive sweating under my armpits, but it occasionally affects my daily life. |
| Level 3 | Sweating under my armpits is almost unbearable and often affects my daily life. |
| Level 4 | Sweating under my armpits is unbearable and always affects my daily life. |

**Table S5. Axillary osmidrosis classification**

| Level 0 | Under no circumstances will the armpit emit a foul odor. |
| --- | --- |
| Level 1 | Only after heavy physical activity (such as exercise) does the armpit emit a slight odor that one can smell. |
| Level 2 | A strong odor emanates from the armpits after certain daily activities, audible from a distance of one meter. |
| Level 3 | When there is no activity, the armpits will also emit a strong odor that is audible within a 1-meter distance. |
